# Disposing of Excess Vaccines After the Withdrawal of Oral Polio Vaccine

**DOI:** 10.1093/infdis/jiw572

**Published:** 2017-06-30

**Authors:** Sarah Wanyoike, Alejandro Ramirez Gonzalez, Samantha B. Dolan, Julie Garon, Chantal Laroche Veira, Lee M. Hampton, Diana Chang Blanc, Manish M. Patel

**Affiliations:** 1 World Health Organization, Geneva, Switzerland;; 2 Global Immunization Division, Center for Global Health, Centers for Disease Control and Prevention,; 3 Division of Infectious Diseases, Emory University School of Medicine, and; 4 Task Force for Global Health, Atlanta, Georgia

**Keywords:** polio, poliovirus, OPV, oral polio vaccine, waste management, disposal.

## Abstract

Until recently, waste management for national immunization programs was limited to sharps waste, empty vaccine vials, or vaccines that had expired or were no longer usable. However, because wild-type 2 poliovirus has been eradicated, the World Health Organization’s (WHO’s) Strategic Advisory Group of Experts on Immunization deemed that all countries must simultaneously cease use of the type 2 oral polio vaccine and recommended that all countries and territories using oral polio vaccine (OPV) “switch” from trivalent OPV (tOPV; types 1, 2, and 3 polioviruses) to bivalent OPV (bOPV; types 1 and 3 polioviruses) during a 2-week period in April 2016. Use of tOPV after the switch would risk outbreaks of paralysis related to type 2–circulating vaccine-derived poliovirus (cVDPV2). To minimize risk of vaccine-derived polio countries using OPV were asked to dispose of all usable, unexpired tOPV after the switch to bOPV. In this paper, we review the rationale for tOPV disposal and describe the global guidelines provided to countries for the safe and appropriate disposal of tOPV. These guidelines gave countries flexibility in implementing this important task within the confines of their national regulations, capacities, and resources. Steps for appropriate disposal of tOPV included removal of all tOPV vials from the cold chain, placement in appropriate bags or containers, and disposal using a recommended approach (ie, autoclaving, boiling, chemical inactivation, incineration, or encapsulation) followed by burial or transportation to a designated waste facility. This experience with disposal of tOPV highlights the adaptability of national immunization programs to new procedures, and identifies gaps in waste management policies and strategies with regard to disposal of unused vaccines. The experience also provides a framework for future policies and for developing programmatic guidance for the ultimate disposal of all OPV after the eradication of polio.

Trivalent oral polio vaccine (tOPV) has been the primary vaccine used worldwide over the past several decades to provide immunity against all 3 types of wild polioviruses (WPVs), known as types 1, 2, and 3. WPV1 still causes cases of polio, but WPV2 was last detected in 1999 and was declared eradicated in September 2015 by the Global Commission for the Certification of the Eradication of Poliomyelitis (GCC), while WPV3 was last detected in November 2012 [1, 2]. Although oral polio vaccine (OPV) has been instrumental toward the interruption of polio transmission, it may, in rare circumstances, cause cases of polio. OPV contains live attenuated poliovirus strains that genetically evolve as they replicate in vaccine recipients and their chains of contacts; those that have mutated during replication may, in very rare circumstances, cause cases of vaccine-associated paralytic polio (VAPP) in vaccine recipients or their close contacts after vaccination [3]. With subsequent transmission through chains of contact, OPV may accumulate sufficient mutations to regain both neurovirulence and transmissibility, resulting in circulating vaccine-derived polioviruses (VDPVs) that can cause outbreaks of paralysis similar to WPV. From January 2006 to May 2016, over 94% of the detected cases of polio caused by cVDPVs and 26%–31% of the VAPP cases were associated with the type 2 component of tOPV [4, 5].

To prevent cases of polio caused by VDPVs as the world moves toward global eradication of poliovirus, the Global Polio Eradication Initiative’s (GPEI’s) Polio Eradication and Endgame Strategy, 2013–2018 (ie, the Endgame Plan) [6] called for a phased withdrawal of OPV [7]. The first phase of the eradication of WPV2 necessitated a “switch” by all countries using OPV from tOPV to bivalent OPV (bOPV), which only contains attenuated types 1 and 3 polioviruses and thus removes type 2 poliovirus vaccine (OPV2) from regular use. The World Health Organization’s (WHO’s) Strategic Advisory Group of Experts (SAGE) on Immunization recommended that all OPV using countries and territories switch from tOPV to bOPV between 17 April to 1 May, 2016 and dispose all excess tOPV as soon as possible after the switch [8]. In addition, SAGE recommended that these countries introduce at least 1 dose of IPV by the end of 2015 to provide additional immunity against all 3 types of polioviruses [4, 5].

The synchronized withdrawal and disposal of tOPV addresses the risk that regions still using tOPV could generate or export new cVDPV2s [9]. The cessation of OPV2 was expected to cause a decrease in population immunity against type 2 poliovirus infections, thus increasing the risk of cVDPV2s emerging or causing outbreaks if populations were exposed to tOPV [9] or a cVDPV2 that has been circulating prior to the switch. A key strategy for avoiding post-switch outbreaks was to maximize population immunity to type 2 poliovirus before the switch. As such, it was necessary for countries to ensure that sufficient tOPV was available before the switch for vaccination campaigns and supplemental immunization activities, and to cease tOPV use immediately after the switch. This strategy inevitably would lead to some amount of excess tOPV after the switch in countries where some 130 million children are born each year [10] and an estimated 2 billion doses were used annually. Preventing intentional or inadvertent tOPV use after the switch through appropriate disposal of remaining tOPV as soon as possible after the switch was important for reducing the risk of post-switch cVDPV2 outbreaks.

Disposal of vaccines can be categorized under the management of health-care waste, or can be classified nationally under the scope of pharmaceutical waste management. Health-care waste includes a broad range of materials, of which a large component can be classified as general nonhazardous waste and a smaller proportion as hazardous [11]. Unused vaccines (eg, expired or no longer needed) constitute a small fraction of the overall burden of health-care waste management. Previously, guidelines for disposal and management of health-care waste focused on the disposal and destruction of needles and syringes, not unexpired vaccine that was not heat damaged [12–15]. Prior to the guidance by GPEI, no guidance had been developed to deal with OPV disposal by vaccination programs in the context of polio eradication. The Immunization Systems Management Group (IMG) of the GPEI, a time-limited entity responsible for the management and coordination of partners’ activities toward achieving Objective 2 of the Endgame Plan, was charged with the task of developing global guidance for the safe and appropriate disposal of tOPV.

In this paper, we review the rationale and global guidance provided by the IMG [16, 17] on possible tOPV waste management strategies (Table 1) for national immunization programs. Experience from the development of these tOPV disposal guidelines and how countries used them may inform future disposal activities for other vaccines, particularly the future withdrawal of all OPVs after the eradication of polio.

**Table 1.** Summary of the Recommended tOPV Inactivation and Disposal Methods

	Autoclaving	Boiling	Chemical Inactivation	Encapsulation	Incineration
Definition in the IMG guidelines	The use of high-pressure steam at 121°C–134°C to kill pathogens over a specified duration	Boiling tOPV vials at water boiling temperature (100°C) for 30 minutes	Immersing tOPV vials in 0.5% chlorine bleach solution for 30 minutes	Immobilization of tOPV vials using impervious material (such as cement) in a container	Controlled burning of tOPV vials in a furnace at temperatures >1100°C for complete combustion
Ideal use	Autoclaving should be done in a large autoclave with integrated shredder; alternatively, vials can be opened and treated in any autoclave	Boil unopened vials	Chemically inactivate opened vials using bleach or other chlorine solution at the recommended concentrations (0.5%)	Encapsulate unopened vials in containers filled with concrete	Incinerate in a high-temperature incinerator capable of safely handling glass (such as a rotary kiln incinerator)
Drawback	Unopened/unshredded vials may not be fully inactivated in an autoclave, especially if the autoclave has been densely packed with other waste that could act as an insulator; closed glass vials may explode under pressure if unopened	Boiling may be impractical for treating large quantities of vials; operators must be careful to avoid scalding	Expensive for processing large quantities of vials, requires operators to be trained in using chlorine solution; chlorine solution must be safely disposed of	Concrete-filled containers must still be securely buried	Melted glass can damage incinerators at temperatures <1100°; closed glass vials can explode under pressure if unopened; plastic vial incineration is prohibited in many countries due to toxic emissions
Disposal	Transport of the waste materials to a waste facility; burial of the waste in a secured and inaccessible pit or landfill				

## KEY CONSIDERATIONS IN PLANNING FOR tOPV DISPOSAL

The stability of OPV and worker safety were 2 key considerations for identifying appropriate tOPV disposal strategies.

### OPV Stability

The need for considering disposal strategies for tOPV after the switch was supported by 2 factors relevant to the stability of tOPV upon removal from the cold chain. First, although OPV is one of the most heat sensitive of all vaccines, it does not lose infectivity immediately after removal from the cold chain [18]. OPV samples exposed to 37°C lose almost complete infectivity gradually over 21 days, but this varies based on viral type, nature of stabilizer, and pH [19]. According to a WHO review, “oral poliomyelitis vaccines may lose 4% to 13% of their activity per day at 25°C, 11% to 21% per day at 31°C, and 26% to 34% per day at 37°C” [19]. OPV titer loss is negligible when stored at 2°C–8°C for up to 6 months or at –20°C for up to 2 years [20]. However, temperatures of 50°C or higher destroy polioviruses quickly [21].

Second, it is important to note that although OPV that has lost half its potency would not meet regulatory standards for use as an effective vaccine, it is still possible that the virus in a vaccine with this or a lower potency could infect an individual and replicate if administered [22, 23]. The lower the potency, the lower the likelihood that the virus in the vaccine could cause an infection. This potential for polio infection, transmission, and replication could pose a risk for generating VDPVs if partially potent tOPV were used after the switch in the context of low population immunity (eg, if tOPV is placed back into the cold chain within 2–3 days of withdrawal and is subsequently administered months after the switch). As such, in addition to removing tOPV from the cold chain, its appropriate disposal would prevent inadvertent or intentional use after the switch [16].

### Worker Safety Considerations

Assuring the safety of the health-care workers was an important consideration for tOPV disposal, as improper disposal could result in release of toxic pollutants or physical injury. Countries were encouraged to plan for and to implement appropriate safety training, provide safe equipment and resources, and establish safety standards for the disposal of tOPV. Examples of types of potential injuries may include scalding injuries from boiling, cuts from broken glass, exposure to toxic fumes while incinerating or burning, and exposure to high concentrations of bleach or potentially caustic encapsulating materials. Although these types of injuries were anticipated hazards for workers dealing with waste beyond just tOPV, the risk may have been higher when dealing with unusually large volumes of waste during the switch. Because of its unprecedented nature, the switch offered many countries and partners an opportunity to evaluate the safety of their work environment, as well as reinforce messages about creating and sustaining them.

## CHALLENGES, GAPS, AND LESSONS LEARNED

The development of prescriptive guidance (ie, guidance with limited flexibility) for tOPV disposal was untenable due to variations in sovereignty of immunization programs, national regulations in countries for handling pharmaceutical waste, country capacity and resources, and anticipated volumes of tOPV for disposal. As such, challenges existed in developing guidance that was pragmatic and implementable, particularly for countries in low-resource settings or in the absence of national legislation. Some of these challenges included: limited experience in disposal of OPVs, difficulty classifying tOPV waste like other health-care and pharmaceutical waste, varying country contexts (eg, capacity and legislation), and limited knowledge on consequence of not disposing tOPV appropriately.

First, in the early stages of the switch planning at the global level, limited expertise existed within GPEI and its partner organizations on vaccine waste management, particularly with regard to disposal of unused vaccines. Although immunization programs have had substantial experience in disposing of vials of vaccines that have been opened, expired, or damaged from heat exposure, they have had less experience with destroying large volumes of potent vaccines. Disposal of tOPV was not perceived to be a complicated task, given that OPV is known to be one of the most heat-sensitive EPI vaccines, thus supporting the notion that removal from the cold chain would render it inactive. However, data from studies on OPV inactivation indicated that OPV does not completely lose potency when stored at 37°C for 2–3 days. Moreover, country concerns about excess tOPV remaining after the switch, increasing requests for guidance on how to dispose of tOPV, and the need to reinforce the importance of not using tOPV after the switch prompted a concerted effort to develop guidelines that described tOPV waste disposal options.

Second, determining whether or not tOPV should be classified as pharmaceutical versus infectious waste may affect local disposal strategies. Classification of health-care waste is the basis for development of waste management policies and regulations, and for selecting the appropriate waste management strategies. According to experts from the International Solid Waste Association (ISWA), the risk classification for tOPV remaining after the switch could not be clearly defined. ISWA highlighted a conclusion from a discussion group in 2003 on global containment strategies that “the assignment of specific biohazard risk classifications to Sabin attenuated poliovirus strains as global immunity wanes should be conducted” [31]. Based on the Basel Convention and WHO’s guidance on safe management of waste from health-care activities, both pharmaceutical or infectious waste classifications may be applicable to OPV [32, 33]. Pharmaceutical waste can be handled through a broader array of waste management options compared to hazardous or nonhazardous infectious waste, thus providing countries with the flexibility to adapt guidance to their situation and capacity. Future guidance on delineating the classification of vaccine waste would be useful for determining appropriate vaccine disposal strategies.

Third, the development of a single, uniform global disposal plan was further challenged by country variations that exist in health-care waste regulations and policies. For example, some national legislation prohibited the destruction of unexpired vaccines, which underscored the need to emphasize that countries should follow country legislation primarily, supplemented by the global guidance for acceptable methods of inactivation and disposal. The development of global guidance was further complicated by variations in national toxic emission regulations that would affect the selection and implementation of disposal strategies. To this end, the identification of core recommendations for tOPV disposal methods provided templates for developing country-specific plans that were adapted to national realities surrounding this topic.

Fourth, several options exist for destroying live virus vaccines such as OPV. Selection and implementation of the appropriate vaccine waste management strategy depends on factors such as country resources (eg, whether a country has incinerators or autoclaves), feasibility (eg, are incinerators available in every district or only at national level), and existing policies and national regulations. Although some national immunization programs have policies, guidelines, and action plans for health-care waste, few address the disposal of usable vaccines. Conversely, some countries were also noted to have laws in place that prohibit the destruction of unexpired (viable) vaccines. An expert review of the pros and cons of vaccine disposal methods and development of normative guidance for future disposal of live virus vaccines would facilitate country preparedness and planning to address this gap in vaccine waste disposal. Moreover, operational research is necessary to address issues related to disposal strategies, such as the need to open vials for autoclaving, duration of boiling, and risk of glass propulsion during autoclaving or incinerating.

Last, gaps exist in the available scientific and practical information for quantifying the risks associated with not appropriately disposing of tOPV after the switch. The rationale for not using the vaccine if it has been exposed to significant amounts of heat is supported by data demonstrating that OPV degrades with exposure to heat. Although evidence exists that far smaller doses of Sabin-strain type 2 polioviruses than what is contained in tOPV (according to WHO standards) can cause infections [22], the exact per-dose risk for generating vaccine-derived viruses from partially potent OPV remains unknown. Even if partially potent tOPV is used after the switch, ongoing transmission of type 2 poliovirus may not occur for multiple reasons, including possible failure by the vaccine virus to infect the recipient, a local environment that is not conducive to the spread of poliovirus, or a local population that has sufficient herd immunity to block person-to-person transmission [9]. Other risks that are difficult to quantify include the possibility that tOPV is returned back to the cold chain before complete inactivation and subsequent inadvertent or intentional use of potent tOPV after the switch. The role that communicating “tOPV must be destroyed” played in motivating health workers to stop tOPV use and remove tOPV from the cold chain at the designated time is similarly difficult to quantify yet important. Further work on risks associated with inappropriate disposal of tOPV would help determine the importance of OPV disposal and the allocation of appropriate resources and strategies to deal with the future bOPV withdrawal after eradication.

## OVERVIEW OF tOPV DISPOSAL GUIDANCE

The IMG’s primary recommendation for the disposal of tOPV was that countries should follow existing national legislation and guidance provided by their pharmaceutical management divisions. For countries that lacked relevant national legislation and guidance, the tOPV disposal guidelines focused on principles of waste management directly relevant to tOPV rather than the broader practice of health-care waste management for immunization programs. tOPV disposal required: (1) inactivation of tOPV, and (2) disposal of vials and their contents in a safe and nonhazardous manner. Steps for appropriate disposal of tOPV included the removal of all tOPV vials from the cold chain, placement in appropriate bags or containers, and inactivation using 1 of the recommended approaches (ie, autoclaving, boiling, chemical inactivation, incineration, or encapsulation). Selection of the appropriate disposal strategy would have had to be dictated by the country realities, following waste management guidance and laws, if applicable. Global guidance was developed to encourage country adaptability, creativity, and leadership, building upon the principles of tOPV disposal that were communicated to countries.

## GLOBAL GUIDANCE ON DISPOSAL OF tOPV

At the outset, the IMG consulted an advisory group from the Environmental Waste Group at WHO and experts from ISWA to identify the relevant tOPV disposal methods. Three key considerations emerged for selecting an appropriate disposal method: volume, vial material, and whether vials were open or closed. A small batch of excess vials (eg, 20 vials or 200–400 tOPV doses) could be disposed of locally at a health facility or district level, and thus might be amenable to approaches already routinely used by health-care staff (eg, boiling, autoclaving) [16]. Vial material was important because some countries had regulations against toxic emissions that resulted from burning plastics and rubber. While tOPV vials in use at the time of the switch were made of glass, the stoppers were made of rubber and plastic, and combustion of large volumes in an incinerator might be inappropriate, depending on national toxic emission regulations. Last, whether the vials were opened or closed was also an important consideration, because closed glass vials might not allow for adequate penetration of inactivation agents (eg, steam or disinfectants) to ensure the complete inactivation of tOPV.

## RECOMMENDED APPROACHES FOR tOPV INACTIVATION

Countries were advised to ensure that the selected destruction approach(es) addressed both inactivation and disposal of tOPV. This may have involved adopting a combination of 2 methods, each dealing with inactivation or disposal (eg, boiling followed by burial; autoclave followed by burial), or a single method that inactivates and disposes (eg, incineration). Each method required that the residual waste (eg, vials, ashes, encapsulated containers) from the inactivation be disposed appropriately according to local waste management policies (eg, burial or landfill).

### Autoclaving

Autoclaving, also known as steam sterilization, is the use of pressurized steam to kill pathogens. Specific temperatures during autoclaving must be reached (121°C–134°C) and maintained to ensure microbicidal activity, even though it is accepted that OPV readily inactivates at a much lower temperature [24]. The duration of autoclaving for a specific load is determined by load size, density, integrity of the container, and amount of residual air and moisture content in the waste [25]. Moist heat destroys microorganisms by the irreversible coagulation and denaturation of enzymes and structural proteins [24]. There were no data found on whether or not steam generated during autoclaving would be sufficient for inactivating tOPV in closed vials and the duration of autoclaving necessary for closed vials.

### Boiling

Several studies have successfully demonstrated that viruses, enteric bacteria, and protozoa in liquids are sensitive to inactivation when exposed to increasing temperatures [26]. For poliovirus, a study in 2002 demonstrated that subjecting the virus in water to temperatures of 55°C for 30 minutes or temperatures of 95°C for 30 seconds completely inactivated polioviruses [27]. Thus, immersing tOPV vials in boiling water for approximately 30 minutes would be more than adequate to inactivate tOPV.

### Chemical Inactivation

Chemical inactivation treatment methods, commonly used in laboratory settings, use chemical disinfectants to destroy pathogens in waste. Efficacy depends on the type of disinfectant used, concentration, and duration of exposure. Polioviruses are readily inactivated by solutions of formaldehyde and free chlorine (bleach) [28], and are resistant to inactivation by common laboratory disinfectants such as alcohol and cresols. WHO recommends 0.5% chlorine bleach as a laboratory disinfectant for poliovirus [29]; thus, immersion of tOPV in 0.5% chlorine solution for at least 30 minutes would be adequate to inactivate tOPV.

### Incineration

Incineration is a high-temperature dry-oxidation process that reduces organic and combustible waste to inorganic incombustible matter, resulting in a significant reduction of waste volume and weight [25]. Because of the high temperatures involved (more than 800°C), incineration met the technical characteristics of both inactivating and disposing of tOPV vials simultaneously. Although high-temperature incineration had many advantages for disposing of tOPV, functioning high-temperatures incinerators were not readily available in many low-income settings. Even if such incinerators were present in a country, using them would require substantial advance notice for considerations such as logistics, transportation, and contracting with facilities that have functioning equipment.

Incineration of tOPV raised 3 concerns. First, incineration at temperatures less than 800°C could lead to partially melted glass vials that could damage the interior of the incinerator. Second, the potential propulsion of glass vials under high temperatures remains to be evaluated. Third, as previously noted, concerns existed that the combustion of the plastic or rubber tops of tOPV vials might release fumes incompatible with national emission standards or environmental regulations.

### Encapsulation

The WHO guidelines for safe disposal of unwanted pharmaceuticals in and after emergencies define encapsulation as the immobilization of pharmaceuticals in a solid block within a plastic or steel container [30]. Typically, the containers are filled up to 75% capacity with solid and semisolid pharmaceuticals, and the contents are then covered with an immobilizing medium (eg, cement, cement and lime mixture, plastic foam, or bituminous sand). Containers are sealed after the medium has dried and solidified. Although encapsulation did not directly inactivate tOPV, it rendered tOPV essentially inaccessible.

## COUNTRY PREPARATIONS FOR tOPV DISPOSAL

All 155 countries and territories that used OPV in 2015 were advised to establish written plans for the safe disposal of tOPV waste after the switch based on the GPEI guidance for implementing the switch [16]. Countries and territories developed national plans to facilitate standardized disposal procedures and needed to ensure that the appropriate disposal methods were utilized on a national scale, considering the ideal use and drawbacks of each of the recommended methods (Table 1). Preparatory activities also included the training of switch support teams and health workers to achieve the acceptable standards for tOPV disposal, and to ensure compliance with national regulations on health-care waste management. The IMG encouraged countries to assess their waste management capacity in terms of equipment and trained staff, to provide necessary disposal materials, to plan for pick-up and transport of excess tOPV, to identify disposal sites, and to establish contracts if needed [16]. The development of operational microplans for the management of tOPV waste at all administrative levels was a critical component of disposal activities. The IMG recommended that countries dispose of their tOPV as soon as possible after the switch—at the latest within 3 months of the global withdrawal of OPV2—in accordance with WHO’s third Global Action Plan (GAPIII) to minimize poliovirus facility-associated risk [34].

## CONCLUSIONS

**Figure 1.**
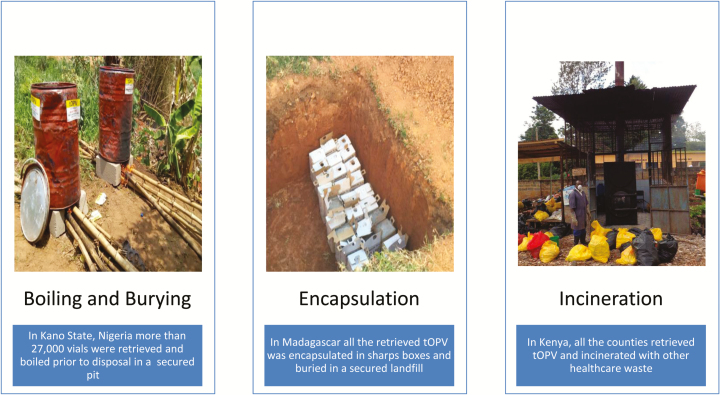
Examples of disposal methods used by various countries.

The lessons learned from the tOPV disposal guidelines and country experiences during the switch provide a framework for future policy discussions around waste management of unused vaccines, and more specifically provide a successful benchmark and platform for planning all OPV withdrawal after the imminent eradication of polio. Our experience highlights that there is no satisfying global consensus around ideal, practical, and environmentally friendly approaches for destroying large volumes of vaccines in resource-poor settings. Countries implemented a variety of the IMG recommended disposal strategies or creative solutions that met the principles of tOPV disposal (Figure 1).

Future efforts to assess the operationalization of the planning and implementation of tOPV disposal in countries would provide valuable lessons and identify best practices that would inform the vaccine disposal policy development and modification of global guidelines for OPV disposal after polio eradication. In addition, the risk classification for the Sabin-attenuated live polioviruses needs to be clearly defined to better inform waste management policies and regulations for OPV. Further information on the risks associated with inappropriate disposal of OPVs would help reinforce future messages on the importance of allocating adequate resources for the future disposal of bOPV and mOPV.

The last mile of polio eradication calls for synchronized management of global vaccine stocks on an unprecedented scale. The eradication of type 2 poliovirus and the global access to bOPV have provided sufficient grist to launch the first phase of the Endgame Plan, which called for OPV2 cessation and ultimately all OPV cessation after complete polio eradication. Containment of type 2 poliovirus—wild, vaccine-derived, and the Sabin/OPV strains—is critical for sustaining the gains of polio eradication and to avoid reintroductions of type 2 viruses as immunity declines after eradication. Discontinuing the programmatic use of tOPV and destroying any residual vaccine were part of the larger strategy to ensure that the world remains free of type 2 polioviruses. The experience gained with the disposal of tOPV will be particularly relevant for bOPV withdrawal after the eradication of types 1 and 3 wild polioviruses.

## Notes

***Financial support.*** This work was supported in part by the Bill and Melinda Gates Foundation (M. M. P., J. G., C. L. V., and S. W.).

***Supplement sponsorship.*** This work is part of a supplement coordinated by the Task Force for Global Health with funding provided by The Bill and Melinda Gates Foundation and the Centers for Disease Control and Prevention.

***Potential conflicts of interest.*** All authors: No reported conflicts.
